# Sixty-Two Years of Internal Mammary Artery Grafting and Forty Years of a Milestone Paper

**DOI:** 10.21470/1678-9741-2026-0098

**Published:** 2026-06-12

**Authors:** Andrea Cristina Oliveira Freitas, Henrique Murad

**Affiliations:** 1 Department of Surgery, Instituto Dante Pazzanese de Cardiologia, São Paulo, São Paulo, Brazil; 2 Universidade Federal do Rio de Janeiro, Hospital Universitário Clementino Fraga Filho, Rio de Janeiro, Rio de Janeiro, Brazil

**Figure:**
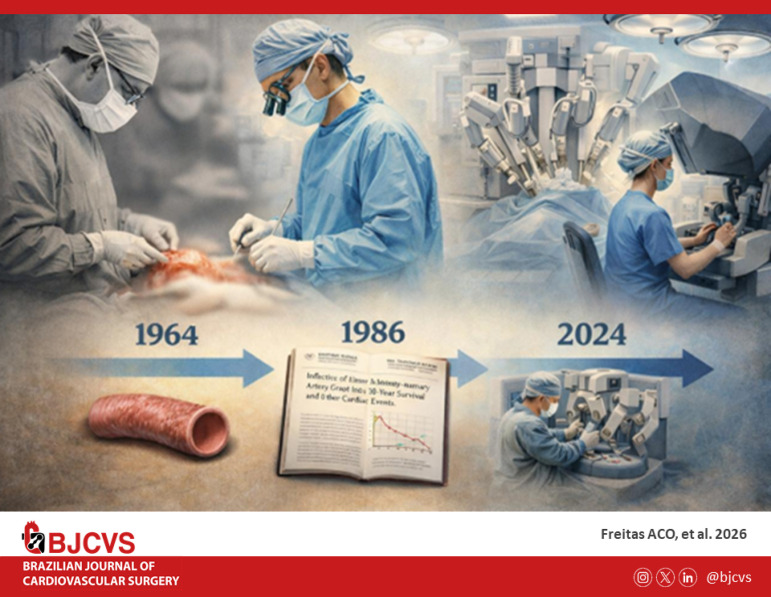

## The Historical Landmark

In 1964, in Leningrad, Vasilii Ivanovich Kolessov performed the first internal mammary artery (IMA)-to-left anterior descending artery (LAD) anastomosis without cardiopulmonary bypass^[1]^. Four years later, in 1968, George E. Green reported the first widely recognized IMA-LAD graft in the Western world, following careful experimental validation^[2]^. Throughout the 1970s, surgeons increasingly adopted bilateral IMA grafting, already suggesting superior long-term durability compared with venous conduits^[3]^.

By the early 1980s, the superiority of the mammary artery was no longer merely anecdotal; it was increasingly supported by institutional experience and emerging evidence. However, it was the landmark 1986 publication by the Cleveland Clinic group, led by Floyd D. Loop and Bruce W. Lytle, that transformed surgical conviction into definitive scientific doctrine^[4]^.

Published in *The New England Journal of Medicine*, the study reported 10-year follow-up data from more than 5,000 patients who had undergone coronary artery bypass grafting (CABG) since 1971. The investigators demonstrated significantly improved survival and reduced major adverse cardiac events among patients receiving an IMA graft to the LAD compared with those treated exclusively with saphenous vein grafts^[4]^. Its enduring impact is reflected in the fact that it has been cited more than 3,600 times in major bibliometric sources, underscoring its foundational role in contemporary coronary surgery^[4]^.

At the time, the saphenous vein had been used almost exclusively as a coronary conduit. Accumulating reports had already described intimal hyperplasia and progressive graft failure, particularly beyond the first postoperative year, with clinically relevant early attrition also recognized. The study by Loop et al. emerged in this context of growing concern regarding the durability of venous grafts. In their acknowledgments, the authors cited the support and encouragement of Eugene Braunwald and John W. Kirklin in disseminating the findings, reflecting early recognition of the study’s potential relevance^[4]^.

Although observational and non-randomized, the investigators achieved, even in the 1980s, a mean follow-up of nearly nine years in more than 5,000 patients using systematic longitudinal surveillance. Follow-up was conducted through structured telephone contact and correspondence, with loss to follow-up limited to only nine patients in each group - an exceptionally low number even by contemporary standards^[4]^.

The use of the IMA in CABG resulted in a marked survival benefit, with > 10% additional patients alive at 10 years in the arterial graft group. This advantage was observed across all anatomical subsets - single-, double-, and triple-vessel disease. Notably, the greater the degree of left ventricular dysfunction at the time of surgery, the greater the absolute benefit associated with IMA use, reaching up to a 15% increase in late survival. The IMA group also experienced fewer major adverse cardiac events and fewer cardiac-related readmissions^[4]^.

Beyond clinical outcomes, Loop et al. reported significantly higher patency rates for the IMA grafted to the LAD at 10 years - 96% *vs.* 81% for saphenous vein grafts. Patency of venous grafts to other coronary territories was lower, reaching 74%^[4]^.

Based on their experience and the progressive adoption of the IMA during the study period, the authors recommended the left IMA as the preferred conduit for significant LAD lesions - helping consolidate a practice that would soon become standard worldwide^[4]^.

## Limitations of the Original Study

Important methodological considerations deserve acknowledgment. Patients with significant left main coronary artery stenosis (> 70%) and those undergoing sequential anastomoses with left IMA were excluded. It is plausible that inclusion of these higher-risk subsets might have further amplified survival differences.

In addition, patients who died during the in-hospital phase were excluded from the final survival analysis. Although in-hospital mortality was reported to be three times higher in the venous graft group (1.73% *vs.* 0.52%), these deaths were not incorporated into the long-term survival curves^[4]^.

The exclusively venous group also exhibited a higher rate of incomplete revascularization (45% *vs.* 36%), a factor that may have partially influenced outcomes.

The relatively low proportion of women in both groups reflects the historical underrepresentation of female patients in surgical studies - a disparity that persists. Women continue to receive fewer arterial grafts and demonstrate higher adjusted mortality after CABG. Emerging data suggest that minimally invasive strategies may mitigate part of this difference^[5,6]^.

## Consolidation in International Guidelines

Over the subsequent four decades, numerous studies confirmed and expanded the original findings, which were progressively incorporated into international guidelines. The 2024 ESC Guidelines for the management of chronic coronary syndromes recommend surgical myocardial revascularization with the left IMA as the conduit of choice for patients with significant left main disease, multivessel disease (particularly in diabetic patients), triple-vessel disease with preserved ventricular function, and singleor double-vessel disease involving the proximal LAD - all with Level of Evidence IA^[7]^.

## Technical Evolution of the Internal Mammary Artery

Loop et al. acknowledged the greater technical complexity of pedicled IMA harvesting compared with saphenous vein preparation, recommending perseverance and meticulous technique^[4]^. Since then, harvesting strategies have evolved considerably.

A recent systematic review and meta-analysis suggested that skeletonized harvesting provides a longer conduit and greater flow while better preserving sternal perfusion, potentially reducing sternal wound complications. Experimental work by Gaudino et al. showed that removal of the endothoracic fascia enhances arterial dilation without compromising endothelial integrity^[8,9]^. No consistent difference in overall survival has been demonstrated between harvesting techniques^[9]^.

Long-term follow-up of the original Cleveland Clinic cohort showed that bilateral internal thoracic artery grafting confers additional survival benefit compared with single IMA use in selected low-risk patients, although at the expense of higher sternal complication rates. The survival advantage became more pronounced beyond 15 years^[10]^.

The IMA has also been safely employed as a free graft - either originating from the aorta or configured as a composite Y-graft - despite limited prospective randomized evidence^[11,12]^.

Both left and right IMAs are central to anaortic coronary revascularization strategies aimed at minimizing aortic manipulation and reducing neurological complications^[13]^.

Minimally invasive coronary surgery has expanded substantially. Single IMA-to-LAD grafting remains a principal indication for mini-thoracotomy or robotic approaches, either as an isolated procedure or as part of hybrid revascularization strategies. Shorter hospital stays and faster recovery have been reported, although economic and anatomical constraints remain important considerations^[5]^.

## Histological Properties, Patency, and Risk Factors

Loop et al. hypothesized that the superiority of IMA might be attributable not only to anatomical proximity to LAD but also to intrinsic biological properties^[4]^. Subsequent research confirmed that IMA exhibits enhanced endothelial nitric oxide production, distinctive histological architecture, and relative resistance to atherosclerosis^[14]^.

IMA has been reported to exhibit higher levels of apolipoprotein C-III and paraoxonase compared with coronary arteries, potentially contributing to plaque prevention. Its intimal layer is rich in heparan sulfate and endothelial nitric oxide synthase, contributing to reduced thrombogenicity. Current research also explores the role of gut microbiota and dysbiosis as potential modulators of endothelial injury in bypass conduits^[15]^.

In the original study, IMA grafts remained patent in 85% to 95% of patients at 10 years. Hypercholesterolemia - particularly elevated low-density lipoprotein cholesterol - and uncontrolled diabetes mellitus adversely affected venous graft patency to a greater extent than progression of native coronary atherosclerosis. An increased risk of sudden death related to venous graft occlusion was also reported from the third postoperative year onward^[4]^.

In 2022, a study reported that 26.1% of patients who continued smoking or experienced frequent postoperative hyperglycemia developed IMA stenosis at 10 years^[16]^. Hyperhomocysteinemia has also been identified as a risk factor for graft dysfunction, including in the IMA, by disrupting the balance between vascular dilation and constriction^[17]^.

In contemporary practice, strict control of cardiovascular risk factors and systematic pharmacological optimization are recommended, including long-term low-dose aspirin therapy, dual antiplatelet therapy in selected cases, beta-blockers, and statins^[7]^. Some studies further suggest that preoperative use of angiotensin-converting enzyme inhibitors and beta-blockers may improve intraoperative endothelial reactivity of the IMA^[18]^.

## Four Decades Later

Forty years after its publication, the study by Loop et al. remains one of the cornerstones of CABG. What initially represented a technically differentiated choice has consolidated into a therapeutic foundation, an international guideline standard, and a platform for surgical innovation.

Contemporary discussions now encompass multiarterial strategies, composite configurations, anaortic approaches, minimally invasive surgery, and the molecular biology of arterial endothelium. IMA has evolved from being merely a superior conduit to becoming a symbol of integration between surgical technique, vascular biology, and evidence-based medicine.

## Artificial Intelligence Usage

The authors declare use of ChatGPT for English language review and illustration creation. The content produced by the artificial intelligence tool was revised and edited by the authors as necessary, and they take full responsibility for the content to be published.

## References

[1] Kolessov VI, Potashow LV. (1965). Surgery of the coronary arteries. Eksp Khir Anesteziol.

[2] Green GE, Stertzer SH, Rappaport EH. (1968). Coronary arterial graft. Ann Thorac Surg.

[3] Green G. (1972). Internal mammary artery-to-coronary artery anastomosis:
three-year experience with 165 patients. Ann Thorac Surg.

[4] Loop FD, Lytle BW, Cosgrove DM, Stewart RW, Goormastic M, Williams GW (1986). Influence of the internal-mammary-artery graft on 10-year
survival and other cardiac events. N Engl J Med.

[5] Welton AJ, Pineda AM, Rogers L, Davierwala PM, Zwischenberger BA (2025). Review of minimally invasive coronary artery bypass
grafting. Eur J Cardiothorac Surg.

[6] Koechlin L, Salikhanov I, Gahl B, Miazza J, Mawad B, Landert N (2025). Sex-specific differences in early occlusion rate after coronary
artery bypass grafting. Ann Thorac Surg.

[7] Vrints C, Andreotti F, Koskinas KC, Rossello X, Adamo M, Ainslie J (2024). 2024 ESC Guidelines for the management of chronic coronary
syndromes. Eur Heart J.

[8] Gaudino M, Toesca A, Nori SL, Glieca F, Possati G. (1999). Effect of skeletonization of the internal thoracic artery on
vessel wall integrity. Ann Thorac Surg.

[9] Shafiq A, Maniya MT, Duhan S, Jamil A, Hirji SA. (2024). Skeletonized versus pedicled harvesting of internal mammary
artery: a systematic review and meta-analysis. Curr Probl Cardiol.

[10] Lytle BW, Blackstone EH, Sabik JF, Houghtaling P, Loop FD, Cosgrove DM. (2004). The effect of bilateral internal thoracic artery grafting on
survival during 20 postoperative years. Ann Thorac Surg.

[11] Navaratnarajah M, Al-Zubaidi FI, Raja SG. (2025). The internal mammary artery: use as a free graft in coronary
artery bypass grafting-evidence, technical considerations and
controversies. Perfusion.

[12] Kikuchi Y, Sakata T, Shimoda T, Fukuhara S, Shimamura J, Pompeu Sa M (2026). Free *vs* in-situ right internal mammary artery as
a conduit in coronary artery bypass surgery: a meta-analysis. Interdiscip Cardiovasc Thorac Surg.

[13] Gomes WJ, Gomes EN, Bertini A, Reis PH, Hossne NA. (2021). The anaortic technique with bilateral internal thoracic artery
grafting-filling the gap in coronary artery bypass surgery. Braz J Cardiovasc Surg.

[14] Bhagat R, Young L, Blackstone EH, Bakaeen FG. (2026). Internal mammary artery grafting-a therapy like no
other. JAMA Surg.

[15] Dicks LMT. (2025). Why are internal mammary (thoracic) arteries less prone to
developing atherosclerosis compared to coronary arteries? Do gut microbiota
play a role? A narrative review. Int J Mol Sci.

[16] Zuo HJ, Nan N, Yang HX, Wang JW, Song XT. (2022). Impact of conventional cardiovascular risk factors on left
internal mammary artery graft disease. Front Cardiovasc Med.

[17] Sun WT, Xue HM, Hou HT, Chen HX, Wang J, He GW (2021). Homocysteine alters vasoreactivity of human internal mammary
artery by affecting the KCa channel family. Ann Transl Med.

[18] Dalaklioglu S, Golbasi I, Ogutman C. (2013). Comparative effects of preoperative angiotensin-converting enzyme
inhibitor, statin and beta-blocker treatment on human internal mammary
artery reactivity in patients with coronary artery disease: a pilot
study. Open Cardiovasc Med J.

